# Benzyl *N*-(1-{*N*′-[(*E*)-2-chloro­benzyl­idene]hydrazinecarbon­yl}-2-hy­droxy­eth­yl)carbamate

**DOI:** 10.1107/S1600536811024895

**Published:** 2011-06-30

**Authors:** Marcus V. N. de Souza, Alessandra C. Pinheiro, Edward R. T. Tiekink, Solange M. S. V. Wardell, James L. Wardell

**Affiliations:** a Fundação Oswaldo Cruz, Instituto de Tecnologia, em Fármacos – Farmanguinhos, R. Sizenando Nabuco, 100, Manguinhos, 21041-250 Rio de Janeiro, RJ, Brazil; b Department of Chemistry, University of Malaya, 50603 Kuala Lumpur, Malaysia; c CHEMSOL, 1 Harcourt Road, Aberdeen AB15 5NY, Scotland; d Centro de Desenvolvimento Tecnológico em Saúde (CDTS), Fundação Oswaldo Cruz (FIOCRUZ), Casa Amarela, Campus de Manguinhos, Av. Brasil 4365, 21040-900 Rio de Janeiro, RJ, Brazil

## Abstract

The mol­ecule of the title compound, C_18_H_18_ClN_3_O_4_, is twisted about the chiral C atom with the dihedral angle between the two amide residues being 87.8 (5)°, but, overall, it can be described as curved, with the benzene rings lying on the same side of the mol­ecule [dihedral angle = 62.8 (4)°]. The conformation about the imine bond [1.294 (7) Å] is *E*. In the crystal, a two-dimensional array in the *ab* plane is mediated by O—H⋯O and N—H⋯O hydrogen bonds as well as C—H⋯Cl inter­actions. The layers stack along the *c*-axis direction, being connected by C—H⋯.π contacts.

## Related literature

For background to the use of l-serine derivatives in anti-tumour therapy, see: Jiao *et al.* (2009 ▶); Yakura *et al.* (2007 ▶). For background to *N*-acyl­hydrazone derivatives from l-serine for anti-tumour testing, see: Pinheiro *et al.* (2010 ▶, 2011*a* ▶,*b* ▶); de Souza *et al.* (2010 ▶); Howie *et al.* (2011 ▶).
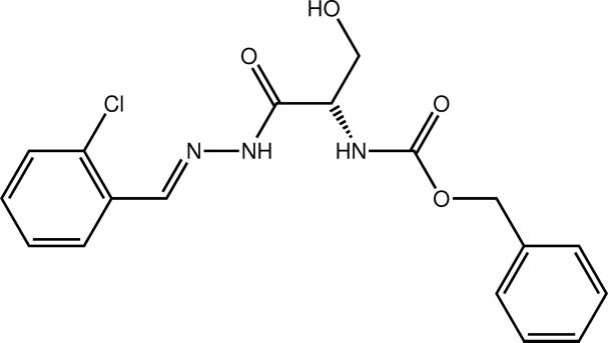


## Experimental

### 

#### Crystal data

C_18_H_18_ClN_3_O_4_
*M* *_r_* = 375.80
Triclinic, 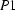

*a* = 4.6804 (4) Å
*b* = 5.6037 (7) Å
*c* = 16.946 (2) Å
α = 95.669 (6)°
β = 95.886 (7)°
γ = 94.467 (6)°
*V* = 438.20 (8) Å^3^
*Z* = 1
Mo *K*α radiation
μ = 0.25 mm^−1^
*T* = 120 K
0.12 × 0.03 × 0.02 mm

#### Data collection

Bruker–Nonius Roper CCD camera on κ-goniostat diffractometer
Absorption correction: multi-scan (*SADABS*; Sheldrick, 2007 ▶) *T*_min_ = 0.682, *T*_max_ = 1.000
6351 measured reflections
3438 independent reflections
2520 reflections with *I* > 2σ(*I*)
*R*_int_ = 0.062

#### Refinement

*R*[*F* ^2^ > 2σ(*F* ^2^)] = 0.070
*wR*(*F* ^2^) = 0.196
*S* = 1.10
3438 reflections
244 parameters
6 restraints
H atoms treated by a mixture of independent and constrained refinement
Δρ_max_ = 0.38 e Å^−3^
Δρ_min_ = −0.39 e Å^−3^
Absolute structure: Flack (1983 ▶), 1476 Friedel pairs
Flack parameter: 0.15 (12)

### 

Data collection: *COLLECT* (Hooft, 1998 ▶); cell refinement: *DENZO* (Otwinowski & Minor, 1997 ▶) and *COLLECT*; data reduction: *DENZO* and *COLLECT*; program(s) used to solve structure: *SHELXS97* (Sheldrick, 2008 ▶); program(s) used to refine structure: *SHELXL97* (Sheldrick, 2008 ▶); molecular graphics: *ORTEP-3* (Farrugia, 1997 ▶) and *DIAMOND* (Brandenburg, 2006 ▶); software used to prepare material for publication: *publCIF* (Westrip, 2010 ▶).

## Supplementary Material

Crystal structure: contains datablock(s) global, I. DOI: 10.1107/S1600536811024895/hb5927sup1.cif

Structure factors: contains datablock(s) I. DOI: 10.1107/S1600536811024895/hb5927Isup2.hkl

Supplementary material file. DOI: 10.1107/S1600536811024895/hb5927Isup3.cml

Additional supplementary materials:  crystallographic information; 3D view; checkCIF report

## Figures and Tables

**Table 1:** Hydrogen-bond geometry (Å, °) *Cg*1 is the centroid of the C13–C18 benzene ring.

*D*—H⋯*A*	*D*—H	H⋯*A*	*D*⋯*A*	*D*—H⋯*A*
O2—H2o⋯O3^i^	0.84 (8)	1.89 (9)	2.728 (7)	171 (9)
N3—H3n⋯O2^ii^	0.88 (6)	2.20 (6)	3.006 (8)	153 (7)
N2—H2n⋯O1^iii^	0.88 (3)	1.93 (4)	2.758 (8)	158 (7)
C6—H6⋯Cl^iv^	0.95	2.81	3.734 (8)	166
C12—H12b⋯*Cg*1^iii^	0.99	2.69	3.474 (8)	137

Symmetry codes: (i) 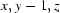; (ii) 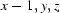; (iii) 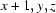; (iv) 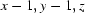.

**Fig. 1.**
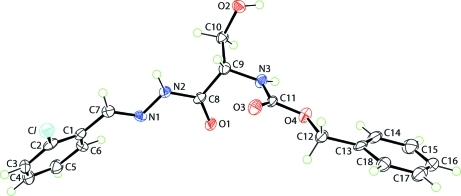
The molecular structure of (I) showing displacement ellipsoids at the 50% probability level.

**Fig. 2.**
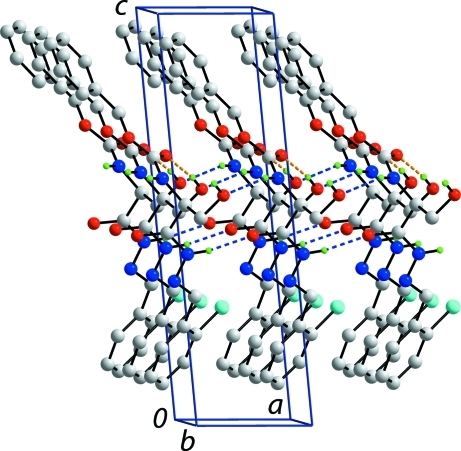
A view of the supramolecular array in the ab plane in (I) with the O—H···O and N—H···O hydrogen bonding shown as orange and blue dashed lines, respectively. Hydrogen atoms not participating in the hydrogen bonding scheme are omitted for reasons of clariy.

**Fig. 3.**
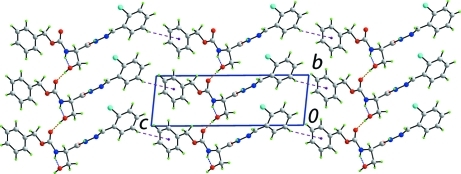
A view in projection down the a axis of the stacking of 2-D supramolecular arrays in the ab plane in (I), and with the O—H···O and N—H···O hydrogen bonding shown as orange and blue dashed lines, respectively.

## supplementary crystallographic information

### Comment

Interest in the development of *N*-acylhydrazone derivatives from *L*-serine for use in anti-tumour testing (Pinheiro *et al.*, 2010; de Souza *et al.*, 2010; Pinheiro *et al.*, 2011*a*: Pinheiro *et al.*, 2011*b*; Howie *et al.*, 2011) arises from the known anti-tumour activity of *L*-serine derivatives (Jiao *et al.*, 2009; Yakura *et al.*, 2007), and motivated the study of the title compound, (I).

Overall, the molecule of (I), Fig. 1, is curved with the benzene rings lying on the same side of the molecule. Nevertheless, the molecule is twisted about the chiral centre with the dihedral angle formed between the two amide residues, *i.e*. N2,C8,O1 and N3,C11,O3,O4, being 87.8 (5) °. The benzyl group is approximately co-planar with the carboxylate group with the dihedral angle between the carbamate group (N3,C11,O3,O4) and benzene ring (C13–C18) being 9.9 (2) °. By contrast, the benzene ring connected to the hydrazine group is twisted out of the plane through the latter as seen in the value of the C2—C1—C7—N1 torsion angle of 146.7 (5) °. The dihedral angle formed between the terminal benzene rings is 62.8 (4) °. The conformation about the N1═C7 imine bond [1.294 (7) Å] is *E*.

The crystal packing is dominated by hydrogen bonding interactions, Table 1. The hydroxyl group forms a O—H···O hydrogen bond with the carbamate-carbonyl group, and simultaneously accepts a hydrogen bond from carbamate-amine. The hydrazine-amine forms a N—H···O hydrogen bond with the carbonyl adjacent to the hydrazine group. The result of the hydrogen bonds is the formation of a two-dimensional array in the *ab* plane, Fig. 2. Additional stabilization to the layer is afforded by C—H···Cl interactions, Table 1. Layers stack along the *c* direction and are connected *via* C—H···π interactions, Table 1 and Fig. 3.

### Experimental

To a stirred solution of methyl (2*S*)-2-[(benzyloxycarbonyl)amino]-3-hydroxypropanoate (0.3 g, 1.17 mmol), prepared from (2*S*)-2-amino-3-hydroxypropanoate hydrochloride and benzyl chloroformate (21 ml, 0.15 mol), in ethanol (10 ml) was added N_2_H_4_.H_2_O (80%, 5.5 mmol). The reaction mixture was stirred for 24 h at room temperature, rotary evaporated and the residue washed with cold ethanol (3 *x* 10 ml) to give benzyl (1*S*)-2-hydrazino-1-(hydroxymethyl)-2-oxoethylcarbamate in 78% yield, which was used as such for the next stage. To a stirred solution of (*S*)-PhCH_2_OCONHCH(CH_2_OH)CONHNH_2_ (1.0 mmol) in ethanol (10 ml) at room temperature was added 2-chlorobenzaldehyde (1.05 mmol). The reaction mixture was refluxed for 4 h, rotary evaporated and the residue purified by washing with cold ethanol (3 *x* 10 ml), affording the title compound, *M*.pt. 438 K, yield 73%.  Yellow needles of (I) for the structure determination were recrystallized from EtOH. ^1^H NMR (500 MHz, DMSO-d6) δ (p.p.m.): 11.79 (1*H*, s, NHN), 8.67 (1*H*, s, N═CH, (*E*)-diastereomer), 7.97 (1*H*, d, J= 6.4, H5), 7.55–7.20 (9*H*, m, Ph, H2, H3, H4 and NHCH), 5.05 (2*H*, s, CH_2_Ph), 4.94 (1*H*, m, OH), 4.15 (1*H*, m, CH), 3.80–3.60 (2*H*, m, CH_2_OH). ^13^C NMR (125 MHz, DMSO-d6) δ (p.p.m.): 172.1, 156.5, 143.5, 137.5, 133.6, 132.0, 131.8, 130.4, 128.8, 128.3, 128.2, 128.1, 127.3, 66.1, 61.9, 57.0. IR (cm^-1^, KBr): 3202 ν(O—H), 1682 ν(COCH and COO). MS/ESI: [M—H]: 374.8.

### Refinement

The C-bound H atoms were geometrically placed (C–H = 0.95–1.00 Å) and refined as riding with *U*_iso_(H) = 1.2–1.5*U*_eq_(C). The O– and N-bound H atoms were located from a difference map and refined with the distance restraints O–H = 0.84 ± 0.01 and N–H = 0.88±0.01 Å, and with *U*_iso_(H) = *zU*_eq_(carrier atom); *z* = 1.5 for O and *z* = 1.2 for N.

### Crystal data

**Table:**

C_18_H_18_ClN_3_O_4_	*Z* = 1
*M**_r_* = 375.80	*F*(000) = 196
Triclinic, *P*1	*D*_x_ = 1.424 Mg m^−^^3^
Hall symbol: P 1	Mo *K*α radiation, λ = 0.71073 Å
*a* = 4.6804 (4) Å	Cell parameters from 13950 reflections
*b* = 5.6037 (7) Å	θ = 2.9–27.5°
*c* = 16.946 (2) Å	µ = 0.25 mm^−^^1^
α = 95.669 (6)°	*T* = 120 K
β = 95.886 (7)°	Needle, yellow
γ = 94.467 (6)°	0.12 × 0.03 × 0.02 mm
*V* = 438.20 (8) Å^3^	

### Data collection

**Table:**

Bruker–Nonius Roper CCD camera on κ-goniostat diffractometer	3438 independent reflections
Radiation source: Bruker-Nonius FR591 rotating anode	2520 reflections with *I* > 2σ(*I*)
graphite	*R*_int_ = 0.062
Detector resolution: 9.091 pixels mm^-1^	θ_max_ = 27.5°, θ_min_ = 3.7°
φ and ω scans	*h* = −6→6
Absorption correction: multi-scan (*SADABS*; Sheldrick, 2007)	*k* = −7→7
*T*_min_ = 0.682, *T*_max_ = 1.000	*l* = −21→21
6351 measured reflections	

### Refinement

**Table:**

Refinement on *F*^2^	Secondary atom site location: difference Fourier map
Least-squares matrix: full	Hydrogen site location: inferred from neighbouring sites
*R*[*F*^2^ > 2σ(*F*^2^)] = 0.070	H atoms treated by a mixture of independent and constrained refinement
*wR*(*F*^2^) = 0.196	*w* = 1/[σ^2^(*F*_o_^2^) + (0.1*P*)^2^] where *P* = (*F*_o_^2^ + 2*F*_c_^2^)/3
*S* = 1.10	(Δ/σ)_max_ < 0.001
3438 reflections	Δρ_max_ = 0.38 e Å^−^^3^
244 parameters	Δρ_min_ = −0.39 e Å^−^^3^
6 restraints	Absolute structure: Flack (1983), 1476 Friedel pairs
Primary atom site location: structure-invariant direct methods	Flack parameter: 0.15 (12)

### Special details

**Table:**

Geometry. All s.u.'s (except the s.u. in the dihedral angle between two l.s. planes) are estimated using the full covariance matrix. The cell s.u.'s are taken into account individually in the estimation of s.u.'s in distances, angles and torsion angles; correlations between s.u.'s in cell parameters are only used when they are defined by crystal symmetry. An approximate (isotropic) treatment of cell s.u.'s is used for estimating s.u.'s involving l.s. planes.
Refinement. Refinement of *F*^2^ against ALL reflections. The weighted *R*-factor *wR* and goodness of fit *S* are based on *F*^2^, conventional *R*-factors *R* are based on *F*, with *F* set to zero for negative *F*^2^. The threshold expression of *F*^2^ > 2σ(*F*^2^) is used only for calculating *R*-factors(gt) *etc*. and is not relevant to the choice of reflections for refinement. *R*-factors based on *F*^2^ are statistically about twice as large as those based on *F*, and *R*- factors based on ALL data will be even larger.

### Fractional atomic coordinates and isotropic or equivalent isotropic displacement parameters (Å^2^)

**Table:**

	*x*	*y*	*z*	*U*_iso_*/*U*_eq_	
Cl	1.3824 (2)	1.3694 (2)	0.28152 (10)	0.0301 (4)	
O1	0.5726 (7)	0.6300 (8)	0.4667 (3)	0.0298 (10)	
O2	1.2666 (8)	0.1671 (7)	0.5712 (3)	0.0270 (10)	
H2O	1.181 (14)	0.079 (11)	0.600 (4)	0.041*	
O3	0.9314 (8)	0.8947 (7)	0.6569 (2)	0.0273 (10)	
O4	0.5835 (8)	0.6630 (7)	0.7041 (2)	0.0265 (9)	
N1	0.8863 (9)	0.7934 (8)	0.3539 (3)	0.0198 (10)	
N2	1.0058 (10)	0.6984 (9)	0.4208 (3)	0.0221 (10)	
H2N	1.195 (3)	0.713 (11)	0.429 (4)	0.026*	
N3	0.8117 (10)	0.4981 (8)	0.6072 (3)	0.0230 (11)	
H3N	0.683 (10)	0.377 (7)	0.610 (4)	0.028*	
C1	0.9669 (12)	0.9974 (11)	0.2410 (4)	0.0233 (13)	
C2	1.1001 (11)	1.2101 (10)	0.2184 (4)	0.0248 (13)	
C3	0.9976 (13)	1.3025 (11)	0.1494 (4)	0.0287 (14)	
H3	1.0844	1.4498	0.1359	0.034*	
C4	0.7682 (13)	1.1799 (12)	0.1000 (4)	0.0315 (15)	
H4	0.6996	1.2419	0.0521	0.038*	
C5	0.6399 (13)	0.9705 (12)	0.1198 (4)	0.0308 (15)	
H5	0.4842	0.8866	0.0851	0.037*	
C6	0.7337 (12)	0.8786 (11)	0.1900 (3)	0.0228 (12)	
H6	0.6394	0.7344	0.2035	0.027*	
C7	1.0712 (12)	0.9037 (10)	0.3152 (3)	0.0223 (12)	
H7	1.2704	0.9233	0.3345	0.027*	
C8	0.8341 (11)	0.6206 (10)	0.4736 (3)	0.0212 (12)	
C9	0.9896 (11)	0.5108 (10)	0.5429 (3)	0.0207 (12)	
H9	1.1710	0.6148	0.5629	0.025*	
C10	1.0689 (11)	0.2567 (11)	0.5148 (3)	0.0218 (12)	
H10A	0.8910	0.1454	0.5048	0.026*	
H10B	1.1540	0.2613	0.4638	0.026*	
C11	0.7900 (11)	0.7019 (10)	0.6551 (3)	0.0190 (11)	
C12	0.5549 (12)	0.8631 (11)	0.7614 (3)	0.0236 (13)	
H12A	0.4789	0.9962	0.7337	0.028*	
H12B	0.7465	0.9217	0.7900	0.028*	
C13	0.3567 (11)	0.7911 (11)	0.8197 (4)	0.0244 (13)	
C14	0.2025 (12)	0.5668 (11)	0.8127 (4)	0.0286 (14)	
H14	0.2219	0.4510	0.7691	0.034*	
C15	0.0196 (14)	0.5110 (13)	0.8693 (5)	0.0373 (16)	
H15	−0.0830	0.3563	0.8648	0.045*	
C16	−0.0126 (13)	0.6770 (13)	0.9310 (4)	0.0363 (16)	
H16	−0.1406	0.6383	0.9688	0.044*	
C17	0.1389 (13)	0.9020 (13)	0.9394 (4)	0.0336 (15)	
H17	0.1162	1.0166	0.9830	0.040*	
C18	0.3217 (12)	0.9584 (11)	0.8845 (4)	0.0275 (13)	
H18	0.4261	1.1127	0.8903	0.033*	

### Atomic displacement parameters (Å^2^)

**Table:**

	*U*^11^	*U*^22^	*U*^33^	*U*^12^	*U*^13^	*U*^23^
Cl	0.0278 (7)	0.0258 (8)	0.0367 (9)	0.0003 (6)	0.0059 (6)	0.0024 (6)
O1	0.017 (2)	0.048 (3)	0.029 (2)	0.0077 (19)	0.0078 (17)	0.016 (2)
O2	0.025 (2)	0.025 (2)	0.034 (3)	0.0034 (18)	0.0036 (18)	0.0113 (19)
O3	0.025 (2)	0.028 (2)	0.028 (2)	−0.0022 (18)	0.0054 (17)	−0.0004 (18)
O4	0.024 (2)	0.031 (2)	0.023 (2)	−0.0005 (18)	0.0069 (17)	−0.0022 (17)
N1	0.020 (2)	0.020 (2)	0.018 (2)	0.0046 (19)	−0.0017 (18)	0.001 (2)
N2	0.017 (2)	0.027 (3)	0.022 (3)	0.0034 (19)	0.0024 (19)	0.005 (2)
N3	0.019 (2)	0.023 (3)	0.028 (3)	−0.0027 (19)	0.008 (2)	0.004 (2)
C1	0.022 (3)	0.029 (3)	0.022 (3)	0.008 (2)	0.009 (2)	0.002 (3)
C2	0.022 (3)	0.028 (3)	0.025 (3)	0.008 (2)	0.008 (2)	−0.007 (3)
C3	0.034 (3)	0.027 (3)	0.031 (3)	0.007 (3)	0.016 (3)	0.012 (3)
C4	0.036 (3)	0.044 (4)	0.017 (3)	0.009 (3)	0.006 (3)	0.010 (3)
C5	0.028 (3)	0.040 (4)	0.025 (3)	0.005 (3)	0.004 (3)	0.004 (3)
C6	0.022 (3)	0.025 (3)	0.023 (3)	0.007 (2)	0.001 (2)	0.009 (2)
C7	0.020 (3)	0.020 (3)	0.025 (3)	0.001 (2)	0.002 (2)	0.001 (2)
C8	0.012 (2)	0.023 (3)	0.027 (3)	−0.001 (2)	0.004 (2)	0.000 (2)
C9	0.015 (2)	0.026 (3)	0.020 (3)	−0.001 (2)	0.001 (2)	0.002 (2)
C10	0.019 (3)	0.028 (3)	0.018 (3)	0.002 (2)	0.002 (2)	0.000 (2)
C11	0.012 (2)	0.027 (3)	0.019 (3)	0.003 (2)	0.004 (2)	0.004 (2)
C12	0.021 (3)	0.029 (3)	0.022 (3)	0.005 (2)	0.005 (2)	0.001 (2)
C13	0.014 (3)	0.035 (3)	0.025 (3)	0.012 (2)	0.003 (2)	0.001 (3)
C14	0.024 (3)	0.035 (4)	0.030 (3)	0.012 (3)	0.008 (3)	0.000 (3)
C15	0.027 (3)	0.035 (4)	0.053 (4)	0.003 (3)	0.013 (3)	0.014 (3)
C16	0.031 (3)	0.053 (4)	0.032 (4)	0.019 (3)	0.014 (3)	0.015 (3)
C17	0.027 (3)	0.047 (4)	0.026 (3)	0.011 (3)	0.003 (3)	−0.003 (3)
C18	0.021 (3)	0.032 (3)	0.028 (3)	0.001 (3)	0.003 (2)	0.000 (3)

### Geometric parameters (Å, °)

**Table:**

Cl—C2	1.739 (6)	C5—H5	0.9500
O1—C8	1.223 (6)	C6—H6	0.9500
O2—C10	1.417 (7)	C7—H7	0.9500
O2—H2O	0.842 (10)	C8—C9	1.525 (8)
O3—C11	1.218 (7)	C9—C10	1.541 (8)
O4—C11	1.357 (6)	C9—H9	1.0000
O4—C12	1.433 (6)	C10—H10A	0.9900
N1—C7	1.294 (7)	C10—H10B	0.9900
N1—N2	1.385 (6)	C12—C13	1.485 (8)
N2—C8	1.343 (7)	C12—H12A	0.9900
N2—H2N	0.877 (10)	C12—H12B	0.9900
N3—C11	1.351 (7)	C13—C14	1.388 (9)
N3—C9	1.441 (7)	C13—C18	1.403 (8)
N3—H3N	0.878 (10)	C14—C15	1.391 (9)
C1—C6	1.400 (8)	C14—H14	0.9500
C1—C2	1.407 (8)	C15—C16	1.357 (10)
C1—C7	1.462 (8)	C15—H15	0.9500
C2—C3	1.381 (8)	C16—C17	1.385 (10)
C3—C4	1.383 (9)	C16—H16	0.9500
C3—H3	0.9500	C17—C18	1.371 (8)
C4—C5	1.364 (9)	C17—H17	0.9500
C4—H4	0.9500	C18—H18	0.9500
C5—C6	1.386 (9)		
			
C10—O2—H2O	111 (5)	N3—C9—H9	108.7
C11—O4—C12	114.7 (4)	C8—C9—H9	108.7
C7—N1—N2	114.6 (4)	C10—C9—H9	108.7
C8—N2—N1	119.6 (4)	O2—C10—C9	112.6 (4)
C8—N2—H2N	124 (4)	O2—C10—H10A	109.1
N1—N2—H2N	115 (4)	C9—C10—H10A	109.1
C11—N3—C9	118.3 (5)	O2—C10—H10B	109.1
C11—N3—H3N	117 (4)	C9—C10—H10B	109.1
C9—N3—H3N	123 (4)	H10A—C10—H10B	107.8
C6—C1—C2	117.8 (5)	O3—C11—N3	127.3 (5)
C6—C1—C7	121.4 (5)	O3—C11—O4	122.9 (5)
C2—C1—C7	120.7 (5)	N3—C11—O4	109.8 (5)
C3—C2—C1	120.9 (5)	O4—C12—C13	110.4 (5)
C3—C2—Cl	119.5 (5)	O4—C12—H12A	109.6
C1—C2—Cl	119.4 (5)	C13—C12—H12A	109.6
C2—C3—C4	119.8 (6)	O4—C12—H12B	109.6
C2—C3—H3	120.1	C13—C12—H12B	109.6
C4—C3—H3	120.1	H12A—C12—H12B	108.1
C5—C4—C3	120.3 (6)	C14—C13—C18	118.4 (5)
C5—C4—H4	119.9	C14—C13—C12	123.1 (5)
C3—C4—H4	119.9	C18—C13—C12	118.5 (5)
C4—C5—C6	120.8 (6)	C13—C14—C15	120.2 (6)
C4—C5—H5	119.6	C13—C14—H14	119.9
C6—C5—H5	119.6	C15—C14—H14	119.9
C5—C6—C1	120.3 (6)	C16—C15—C14	120.2 (7)
C5—C6—H6	119.9	C16—C15—H15	119.9
C1—C6—H6	119.9	C14—C15—H15	119.9
N1—C7—C1	118.5 (5)	C15—C16—C17	120.8 (6)
N1—C7—H7	120.7	C15—C16—H16	119.6
C1—C7—H7	120.7	C17—C16—H16	119.6
O1—C8—N2	123.7 (5)	C18—C17—C16	119.6 (6)
O1—C8—C9	121.7 (5)	C18—C17—H17	120.2
N2—C8—C9	114.6 (4)	C16—C17—H17	120.2
N3—C9—C8	110.6 (4)	C17—C18—C13	120.8 (6)
N3—C9—C10	109.7 (5)	C17—C18—H18	119.6
C8—C9—C10	110.2 (5)	C13—C18—H18	119.6
			
C7—N1—N2—C8	167.6 (5)	N2—C8—C9—N3	163.1 (5)
C6—C1—C2—C3	2.1 (8)	O1—C8—C9—C10	102.9 (6)
C7—C1—C2—C3	−178.3 (5)	N2—C8—C9—C10	−75.4 (6)
C6—C1—C2—Cl	177.8 (4)	N3—C9—C10—O2	−70.8 (5)
C7—C1—C2—Cl	−2.6 (7)	C8—C9—C10—O2	167.1 (4)
C1—C2—C3—C4	−2.6 (8)	C9—N3—C11—O3	−9.6 (8)
Cl—C2—C3—C4	−178.3 (5)	C9—N3—C11—O4	171.2 (5)
C2—C3—C4—C5	1.1 (9)	C12—O4—C11—O3	−3.2 (7)
C3—C4—C5—C6	0.9 (10)	C12—O4—C11—N3	176.0 (4)
C4—C5—C6—C1	−1.4 (9)	C11—O4—C12—C13	−171.5 (5)
C2—C1—C6—C5	−0.1 (8)	O4—C12—C13—C14	−6.0 (7)
C7—C1—C6—C5	−179.7 (5)	O4—C12—C13—C18	174.6 (5)
N2—N1—C7—C1	176.4 (5)	C18—C13—C14—C15	−0.4 (8)
C6—C1—C7—N1	−33.8 (8)	C12—C13—C14—C15	−179.8 (6)
C2—C1—C7—N1	146.7 (5)	C13—C14—C15—C16	1.0 (10)
N1—N2—C8—O1	−0.6 (8)	C14—C15—C16—C17	−1.1 (10)
N1—N2—C8—C9	177.6 (5)	C15—C16—C17—C18	0.5 (10)
C11—N3—C9—C8	−77.4 (6)	C16—C17—C18—C13	0.2 (9)
C11—N3—C9—C10	160.8 (5)	C14—C13—C18—C17	−0.2 (8)
O1—C8—C9—N3	−18.6 (7)	C12—C13—C18—C17	179.3 (5)

### Hydrogen-bond geometry (Å, °)

**Table:**

Cg1 is the centroid of the C13–C18 benzene ring.

**Table:**

*D*—H···*A*	*D*—H	H···*A*	*D*···*A*	*D*—H···*A*
O2—H2o···O3^i^	0.84 (8)	1.89 (9)	2.728 (7)	171 (9)
N3—H3n···O2^ii^	0.88 (6)	2.20 (6)	3.006 (8)	153 (7)
N2—H2n···O1^iii^	0.88 (3)	1.93 (4)	2.758 (8)	158 (7)
C6—H6···Cl^iv^	0.95	2.81	3.734 (8)	166
C12—H12b···Cg1^iii^	0.99	2.69	3.474 (8)	137

Symmetry codes: (i) *x*, *y*−1, *z*; (ii) *x*−1, *y*, *z*; (iii) *x*+1, *y*, *z*; (iv) *x*−1, *y*−1, *z*.
